# Malaria in Skin Diseases—A Correction

**Published:** 1882-12

**Authors:** Lunsford P. Yandell


### Malaria in Skin Diseases—A Correction.

Messrs. Editors:—Some time since, the following paragraph appeared in the *Michigan Medical News*, and has been widely copied in the medical journals of the country:

> A century ago John Hunter divided all skin diseases into three classes, one of which is cured by mercury and the iodides, a second by sulphur, and a third class which the devil himself can't cure. Dr. L. P. Yandell, who quotes Hunter as above, is given credit for a much less complex classification than even this. He attributes all skin eruptions to malaria. Quinine is a specific for malaria; ergo, quinine is the remedy for all skin eruptions. Q. E. D.

I trust that my confreres of the press will do me the kindness and the justice to publish the correction now given, as the matter is not only one of personal interest to the writer, but is of scientific interest to the profession. The subjoined extracts are from a supplement to a report read to the American Dermatological Association, September, 1877. A copy of this report will be gladly sent to any one desiring it:

"From the criticisms which have been made on my views, I find that I have not succeeded in making myself perfectly understood. What I have contended for, and what I have reiterated, is simply this: Malaria is *the chief source* of *acute* skin disease. Scrofula is *the chief source* of *chronic* skin disease. The more inveterate cases of skin disease are often due to the coexistence of these two things. The specific exanthems, of course, are not included here, but I contend that their progress and termination are often largely influenced by the presence of malaria, or struma. *I do not claim* that malaria and struma are the *sole* causes of the dermatoses. Indeed, *many* of the dermatoses may exist *independently* of *malaria* or *struma*, and most frequently some exciting cause is necessary to develop the cutaneous eruption. Among the exciting causes are irritants, injuries, insufficient or improper ingesta, vicissitudes of temperature, alcohol, dentition, menstruation, parturition, lactation, etc. The proofs of the truth of my views are, in the first place, that the diseases of the skin are cured more certainly and more quickly by the anti-malarial remedies on the one hand, and by the anti-strumous on the other, than can be done by any other line of therapeutics; and in the second place, that careful and painstaking investigation will, in the majority of dermatoses, make apparent the existence of the malaria or the struma, as the case may be.

"In conclusion, I desire to impress upon the reader that my views *are not confined to the skin diseases.* What produces disease here will produce it in all other organs of the body.

What is true of dermatology is equally true of gynecology and ophthalmology and otology, and it is just as true of the diseases of all the other regions of the body."

Subsequent observation has confirmed my belief in the correctness of these views. LUNSFORD P. YANDELL.

